# Ecological and Genetic Barriers Differentiate Natural Populations of *Saccharomyces cerevisiae*

**DOI:** 10.1093/molbev/msv112

**Published:** 2015-05-06

**Authors:** Katie J. Clowers, Justin Heilberger, Jeff S. Piotrowski, Jessica L. Will, Audrey P. Gasch

**Affiliations:** ^1^Laboratory of Genetics, University of Wisconsin–Madison; ^2^Great Lakes Bioenergy Research Center, Madison, WI

**Keywords:** ecological divergence, environmental stress, genetic incompatibilities, reproductive isolation, quantitative trait mapping, speciation

## Abstract

How populations that inhabit the same geographical area become genetically differentiated is not clear. To investigate this, we characterized phenotypic and genetic differences between two populations of *Saccharomyces cerevisiae* that in some cases inhabit the same environment but show relatively little gene flow. We profiled stress sensitivity in a group of vineyard isolates and a group of oak-soil strains and found several niche-related phenotypes that distinguish the populations. We performed bulk-segregant mapping on two of the distinguishing traits: The vineyard-specific ability to grow in grape juice and oak-specific tolerance to the cell wall damaging drug Congo red. To implicate causal genes, we also performed a chemical genomic screen in the lab-strain deletion collection and identified many important genes that fell under quantitative trait loci peaks. One gene important for growth in grape juice and identified by both the mapping and the screen was *SSU1*, a sulfite-nitrite pump implicated in wine fermentations. The beneficial allele is generated by a known translocation that we reasoned may also serve as a genetic barrier. We found that the translocation is prevalent in vineyard strains, but absent in oak strains, and presents a postzygotic barrier to spore viability. Furthermore, the translocation was associated with a fitness cost to the rapid growth rate seen in oak-soil strains. Our results reveal the translocation as a dual-function locus that enforces ecological differentiation while producing a genetic barrier to gene flow in these sympatric populations.

## Introduction

Incipient speciation occurs when barriers restrict mating and gene flow between two populations, which can eventually diverge in form and function. In some cases, physical barriers between groups of individuals can lead to reproductive isolation, for example, Darwin’s finches inhabiting different Galapagos islands (Darwin 1859). In other cases, reproductive isolation can occur even when species inhabit the same geographical region in sympatry. The mechanisms underlying sympatric speciation are less clear, because populations have the potential to interact physically. One model is that ecological barriers within the same geographical region create separate niches that impose divergent selective pressures, forcing populations to diverge (Smith 1966). A classic example of sympatric speciation is cichlid fishes, where different populations occupy different ecological niches in the same lake, thereby restricting gene flow and promoting the emergence of separate species over time (reviewed in Fan et al. 2012). Genetic barriers can further promote speciation by creating pre- or postzygotic barriers to reproduction. Despite documented examples, relatively few models for sympatric speciation exist. Studying the process is particularly challenging, as it requires finding subpopulations that are in the early stages of isolation.

Natural populations of budding yeast, *Saccharomyces cerevisiae*, represent an emerging model for studying speciation. There have been many studies on the ecological, genetic, and phenotypic diversity of the species, facilitating experimentation to examine what underlies diversity observed in nature. Population-genomic studies of *S. cerevisiae* have shown a complex pattern of differentiation, with at least ten lineages found in North America, Malaysia, Asia, West Africa, Europe, New Zealand, Israel, and China, along with mosaic strains that represent infrequent out-crossing between lineages (Liti et al. 2009; Schacherer et al. 2009; Wang et al. 2012; Cromie et al. 2013). The distinct lineages partly correlate with geography, environmental niche, and the degree of human association (Fay and Benavides 2005a; Diezmann and Dietrich 2009; Liti et al. 2009; Sicard and Legras 2011; Wang et al. 2012; Hyma and Fay 2013). Phenotypic studies of the species have demonstrated significant variation in phenotypes such as stress tolerance, sporulation efficiency, mRNA and protein levels, and metabolic propensity, some of which vary by lineage and others that are correlated with strain niche (Gerke et al. 2006; Kvitek et al. 2008; Liti et al. 2009; Ehrenreich et al. 2010, 2012; Will et al. 2010; Cubillos et al. 2011; Magwene et al. 2011; Parts et al. 2011; Warringer et al. 2011; Hodgins-Davis et al. 2012; Skelly et al. 2013).

Interestingly, some natural populations of *S. cerevisiae* have been found to co-occur in nature but show only low levels of gene flow (Hyma and Fay 2013). Hyma and Fay (2013) performed a large sampling of strains isolated from tress versus fruit from the same vineyards in Washington and Oregon, USA. They found that strains of the so-called North American “oak” arboreal lineage are generally found on the trees and surrounding soil but occasionally comingle with the vineyard lineage, which is primarily found on fruit but sometimes on the trees. Despite the shared physical association, Hyma and Fay (2013) showed relatively low gene flow between the strains (with the exception of rare hybrids isolated from a particular environment in Northern Wisconsin [Clowers KJ, Will JL, Gasch AP, unpublished data]). Why these comingling populations show little gene flow is not clear.

To provide insight on this question, we conducted a phenotypic and genetic dissection of the North American oak and vineyard populations, by focusing on a collection of naturally isolated strains from each lineage. We find that the populations are distinguished by differences in tolerance to a number of stresses that correlate with differences in the strains’ niches. We conducted a bulk segregant mapping study on two traits that distinguish oak and vineyard strains: Growth in grape juice and resistance to cell wall stress induced by Congo red treatment.

Remarkably, one of the loci underlying growth in grape juice involves a translocation that also presents a cost to an oak-related phenotype and provides significant genetic barrier, by reducing spore viability in crosses between oak and vineyard strains. Our results suggest that a combination of ecological and genetic barriers, in part controlled by a single pleiotropic locus, may be promoting divergence between these sympatric populations.

## Results

### Ecologically Relevant Phenotypes Distinguish Oak and Vineyard Populations

To better characterize phenotypic differences between oak and vineyard populations, we assayed cell viability in a panel of oak and vineyard strains (supplementary table S1, Supplementary Material online), several of which were collected from the same area, as cells grew under a range of stressful conditions (supplementary table S2, Supplementary Material online). These conditions were chosen based on hypothesized differences in the strains’ respective niches and also based on prior results (Kvitek et al. 2008).

**Fig. 1.**
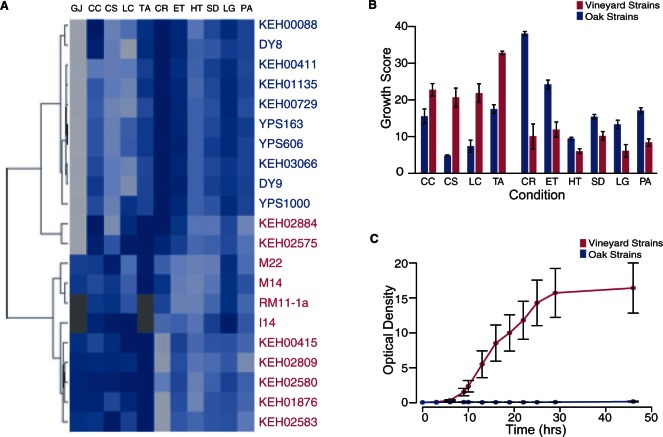
Ecologically relevant phenotypes distinguish oak and vineyard populations. (*A*) Oak (blue, *N* = 10–11) and vineyard (pink, *N* = 12–16 depending on condition) strains were organized by hierarchical clustering based on average phenotype scores in stress compared with YPD control (blue boxes). Each row indicates a strain labeled on the right, and each column represents a different stress condition labeled on the top (CC, cadmium chloride; CS, copper sulfate; LC, lithium chloride; TA, tartaric acid; CR, Congo red; ET, ethanol; HT, heat; SD, sodium dodecyl sulfate; LG, low glucose; PA, potassium acetate). Colored boxes represent the averaged growth score, where dark blue represents rapid growth and light gray indicates no growth under the designated conditions (dark gray indicates missing data). (*B*) The average and standard error of the population mean (SEM) of the oak (blue) or vineyard (pink) phenotype scores represented in (*A*). All of the phenotypic differences shown were statistically significant (*P* < 10^−4^, two-tailed *T*-test with permutation). Caffeine, calcium chloride, and malic acid (not shown) did not significantly distinguish oak and vineyard strains. (*C*) Average and SEM of the optical density (OD_600_) readings for oak (blue) and vineyard (pink) strains grown in white grape juice over 46 h.

We found several phenotypes that distinguish the two populations. As a group, vineyard strains are more tolerant to conditions specific to grapes. These include high osmolarity, copper sulfate (a common antimicrobial agent in vineyards), and tartaric acid, which is abundant in grapes (Jackson 2000)—but not malic acid adjusted to the same pH (fig. 1*A* and *B*). Some of these phenotypic differences were previously reported in the context of other studies (Kvitek et al. 2008; Liti et al. 2009; Will et al. 2010; Cubillos et al. 2011; Warringer et al. 2011). In nature, yeast are not exposed to a single stress at a time, but rather exist in complex niches in which many stressful conditions occur together. To examine a condition that more closely mimics the vineyard environment as a whole, we tested strains for their ability to grow in white grape juice and found that only strains isolated from vineyards were able to grow in this medium (fig. 1*C*). Interestingly, two of the vineyard strains display some phenotypes that are more similar to oak strains (fig. 1*B*). Nonetheless, these results indicate that most vineyard strains are better equipped to survive conditions found in the vineyard environment, including growth in the presence of multiple stresses found associated with grapes.

In contrast, oak strains are more tolerant to a distinct set of stresses. Oak strains grow better on low glucose levels and on nonfermentable carbon sources (fig. 1*A* and *B*), consistent with the idea that they may grow on different or more variable carbon sources in nature. Oak strains are also more tolerant to stresses that affect the cell wall and membrane structure, including Congo red (which binds chitin in yeast cell wall), ethanol, sodium dodecyl sulfate (SDS) detergent, and heat (fig. 1*A* and *B*), although the significance of this is not clear. The differences in tolerance, some of which clearly relate to their unique niches, suggest that strains may have evolved to thrive in different local niches despite a common geographical location.

### Niche-Specific Stress Tolerances Are Complex Traits Linked to Many Genetic Loci

To dissect the genetic basis for phenotypic differences between oak and vineyard strains, we used bulk-segregant mapping to identify loci contributing to growth in grape juice or resistance to Congo red. To capture lineage-level differences, we mapped the phenotypes in two different oak-vineyard crosses. After engineering strain backgrounds for bulk segregant analysis, we mated one of two oak strains, YPS163 or YPS1000 isolated from Pennsylvania and New Jersey, respectively (Sniegowski et al. 2002), to vineyard strains KEH02580 or M14 isolated from vineyards in Missouri (Hyma and Fay 2013) and Italy (Mortimer 2000), respectively. Despite differences in their provenance, the strains genetically represent the oak and vineyard lineages, reflecting that populations in this species are often correlated with niche instead of geography (Fay and Benavides 2005a, 2005b; Aa et al. 2006; Liti et al. 2009; Schacherer et al. 2009; Hyma and Fay 2013). The two crosses generated for this analysis were YPS163 × KEH02580 (Cross 1) and YPS1000 × M14 (Cross 2) (see supplementary table S1, Supplementary Material online).

We first sequenced the genomes of the engineered parental strains. Despite being from the same lineage, the two oak strains show higher levels of polymorphism from each other than the two vineyard strains isolated from different continents. For example, the two oak strains are 0.37% polymorphic from each other (∼46,000 single nucleotide polymorphisms [SNPs]), whereas the two vineyard strains are only 0.086% polymorphic (∼10,000 SNPs). The low rate of polymorphism between the two vineyard strains is consistent with the lineage as a whole, which may be influenced by human-associated migration and domestication (Fay and Benavides 2005a, 2005b; Schacherer et al. 2009; Hyma and Fay 2013). In contrast, the oak and vineyard strains are 0.55–0.57% divergent from each other (∼68,000–71,000 SNPs).

We then performed bulk segregant analysis on two traits: The multistress, vineyard-associated survival in grape juice, as well as the single-stress, oak-specific resistance to Congo red. Large, stable segregant pools (∼10^6^ MAT**a** spores) were generated from each cross and then used to inoculate liquid grape juice or plated on solid medium containing a high dose of Congo red at which approximately 0.5% of spores survived. Segregant pools were sequenced before and after growth under the selective conditions. We then compared frequencies of oak and vineyard alleles after selection with the allele frequencies of the starting pool before selection, using the program MULTIPOOL (Edwards and Gifford 2012) to identify quantitative trait loci (QTL). Results were similar to when the postselection pool was compared with a pool of segregants grown in parallel on no-stress controls (supplementary table S3, Supplementary Material online).

**Fig. 2.**
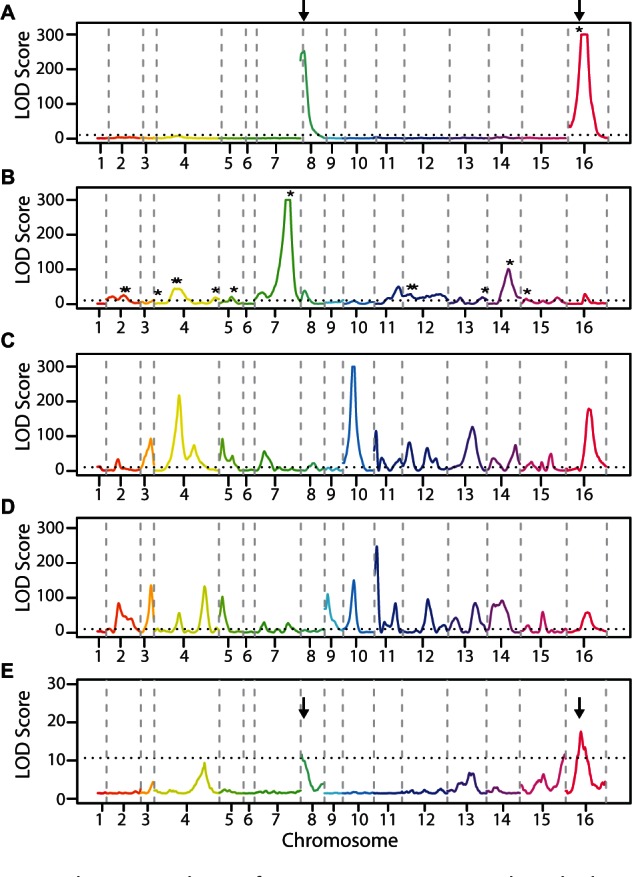
The genetic basis of stress resistances. QTL plots displaying MULTIPOOL LOD scores across all 16 chromosomes for tolerance to grape juice (*A* and *B*), Congo red (*C* and *D*), and rich YPD medium (*E*) in Cross 1 (*A*, *C*, and *E*) and Cross 2 (*B* and *D*). Each chromosome is plotted in a different color and gray vertical dashed lines mark chromosomal boundaries. Black horizontal line shows LOD 10.68 threshold for significance. Asterisks (*) in (*A*) and (*B*) show locations of chemical genomic hits that fall within 90% credible intervals for identified QTL. Arrows show the breakpoints of the Chr 8:16 translocation, see text for details.

The results implicated a total of 65 QTL across the two phenotypes and crosses (summarized in supplementary table S4, Supplementary Material online). The loci associated with grape juice tolerance were surprisingly different between the two crosses (fig. 2*A* and *B*). In Cross 1, we identified two QTL of very large effect, and in both cases the vineyard allele was the beneficial genotype. However, in Cross 2 we found 18 QTL, with a few of large effect and many of smaller effect; the vineyard allele was beneficial at the majority (76.5%) of QTL. In contrast to the case of grape juice, the genetic architecture of Congo red resistance was similar in the two crosses (fig. 2*C* and *D*). Both had a large number of QTL (21 and 24 for Cross 1 and Cross 2, respectively), with almost half (nine) shared by the two crosses. Also in contrast to the grape juice, half of the Congo red QTL (53.3%) showed that the vineyard parent provided the beneficial allele (see Discussion). The results were strikingly different from a control experiment in which the segregants from Cross 1 were grown in rich medium, where there were only three peaks of marginal significance (fig. 2*E*, see more below).

### Chemical-Genomic Screening Implicates Additional Causal Genes for Grape Juice Growth

We leveraged the yeast deletion library generated in the lab (BY background) strain to implicate other genes important for growth in grape juice. The *S. cerevisiae* MAT**a** version of the haploid deletion collection (Andrusiak 2012) was grown in grape juice, and deletion strains with a statistically significant growth difference from control medium (Yeast extract-Peptone-Dextrose [YPD]) were identified. Using this chemical genomics screen, we identified 103 genes in the lab strain that significantly affect fitness in grape juice (false discovery rate, FDR < 0.05; supplementary table S5, Supplementary Material online). Of the 103 genes, 66 caused a fitness defect in grape juice when deleted from the parental strain. These were enriched for genes involved in mitochondria-nucleus signaling (*P* < 2 × 10^−^^5^, hypergeometric test), cellular amino acid biosynthesis (*P* < 5 × 10^−^^5^), regulation of translation elongation (*P* < 1 × 10^−^^4^), retromer complex (*P* < 1 × 10^−^^4^), and signaling proteins that regulate growth rate and carbohydrate metabolism (*P* < 4 × 10^−^^5^). The 37 gene deletions that significantly increase fitness in grape juice are enriched for genes involved in the establishment of cell polarity (*P* < 8 × 10^−^^5^), including key regulators in the cell wall-integrity pathway. When pooled together, the total 103 gene set was enriched for genes involved in lipid catabolic processes (*P* < 2 × 10^−^^5^), implicating membrane function.

Equipped with a better understanding of the physiological response of yeast to grape juice, we tested whether any of the genes identified in the chemical genomics screen were within detected QTL intervals. Of the 103 genes, 15 were within the 90% credible interval of the grape juice QTLs (fig. 2*A* and *B*, asterisks and supplementary table S5, Supplementary Material online), although we note that this could occur by chance (*P* = 0.18, random sampling). Nonetheless, it identifies candidate genes under QTL peaks that may contribute to the phenotypic difference.

### Expression of *SSU1* Underlies the Genetic Basis of Grape Juice Tolerance

We were particularly interested in one gene required for grape juice survival in the lab strain*. SSU1* is a plasma membrane sulfite/nitrate pump (Park and Bakalinsky 2000; Sarver and DeRisi 2005; Mendes-Ferreira et al. 2010; Cabrera et al. 2014) that falls under the large-effect QTL on Chromosome XVI (Chr 16) in Cross 1. This gene has been linked to increased sulfite resistance (Goto-Yamamoto et al. 1998; Park and Bakalinsky 2000) as well as increased wine fermentation rates (Yuasa et al. 2005; Nardi et al. 2010; Brion et al. 2013; Zimmer et al. 2014) and sulfur assimilation (a phenotype important to wine-yeast domestication) (Mendes-Ferreira et al. 2010). Expression of *SSU1* is known to be elevated in several wine strains, due to a reciprocal translocation between Chr 8 and Chr 16 that creates new transcription-factor binding sites in the *SSU1* promoter and increases expression (Pérez-Ortín et al. 2002). The Chr 8:16 translocation has been documented in many industrial wine strains (Yuasa et al. 2004) and is thought to provide an advantage to wine making-strains, in part because sulfur dioxide is added to grape juice as a preservative (Pérez-Ortín et al. 2002; Yuasa et al. 2004, 2005; Nardi et al. 2010; Brion et al. 2013). In fact, the two major-effect QTL identified in Cross 1 lie at the chromosomal breakpoints of this translocation. We confirmed, first by diagnostic polymerase chain reaction (PCR) and then by sequencing, that vineyard strain KEH02580 used in Cross 1 has the translocation.

**Fig. 3.**
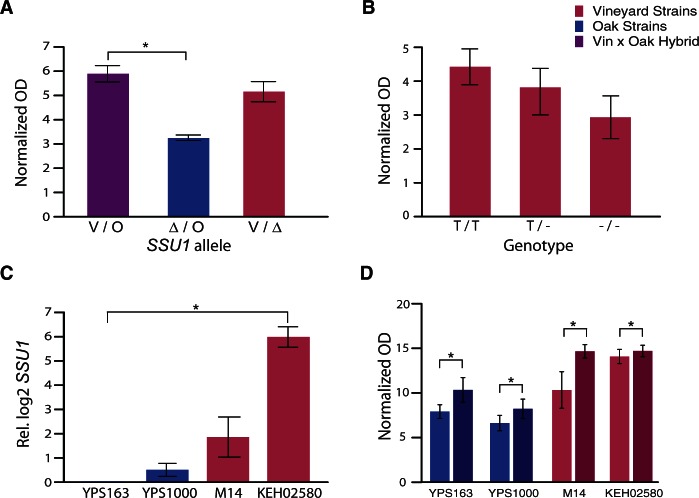
*SSU1* at the Chr 8:16 translocation contributes to growth in grape juice. (*A*) The vineyard allele of *SSU1* is responsible for grape juice QTL. The bars show average final OD_600_ (normalized to the starting OD_600_) for hybrid and hemizygous strains containing different *SSU1* alleles grown in grape juice. Error bars depict standard deviation. Asterisks (*) denote statistical significance (*P* = 0.04, *t*-test). (*B*) Chr 8:16 translocation copy number correlates with grape juice growth in wild vineyard strains. The average final OD_600_ (normalized to the starting OD_600_) for strains grown in grape juice, averaged over vineyard strains homozygous for the translocation (T/T, *N* = 5), heterozygous for the translocation (T/-, *N* = 4), and without the translocation (‐/‐, *N* = 6). Error bars depict the standard error of the mean. (*C*) *SSU1* is highly expressed in KEH02580. Average and standard deviation of *SSU1* transcript abundance (measured in biological duplicate) in each strain normalized to YPS163. Asterisk (*) denotes statistical significance (*P* < 0.2, *t*-test). (*D*) Overexpression of *SSU1* enhances growth in grape juice. Final optical density (OD_600_) was normalized to the starting OD_600_ for each parental strain (*y* axis) overexpressing the vineyard allele of *SSU1* (dark bars) or harboring an empty vector control (light bars). Error bars depict ± standard deviation. Asterisks (*) denote statistical significance (*P* < 0.03, *t*-test).

We confirmed that *SSU1* was the responsible locus, by performing a reciprocal hemizygosity analysis. The hemizygous hybrid strain harboring only the vineyard allele of *SSU1* grew equally well in grape juice as the heterozygous hybrid strain (*P* = 0.1, fig. 3*A*). However, the hemizygous strain with only the oak allele of *SSU1* grew significantly poorer than the hybrid (*P* = 0.4), validating that the KEH02580 vineyard allele of *SSU1* is indeed the causal locus in this cross. Because this allele is linked to the Chr8–Chr16 translocation, we assessed the frequency of the translocation in natural populations. We surveyed a panel of oak and vineyard strains from across the globe for the vineyard allele, by diagnostic PCR and sequencing of the translocation. None of the 12 oak strains surveyed harbored the translocation. Of the 16 wild vineyard strains, 9 had the translocation: 5 strains were homozygous and 4 strains were heterozygous for the translocation. We further examined the correlation between presence of the translocation and the ability of vineyard strains to grow in grape juice. There was a clear trend (although not statistically significant due to several outlier strains, *P* = 0.09, ANOVA [analysis of variance]) between the number of translocation alleles and the final cell density in grape juice, as strains homozygous for the translocation grew better than heterozygotes, which grew better than strains with no translocation (fig. 3*B*). This provides additional support that the *SSU1* allele linked to the translocation contributes to the ability to grow in grape juice.

Somewhat surprisingly, the vineyard strain M14 used in Cross 2 also has the translocation and the same *SSU1* coding sequence, even though the region did not contribute strongly to the phenotype in this cross (fig. 2*B*). Strains are known to vary in the number of a 76-bp repeat found in the promoter of the translocated *SSU1*: more repeats correlates with further increase *SSU1* expression and sulfite resistance (Goto-Yamamoto et al. 1998; Park and Bakalinsky 2000; Pérez-Ortín et al. 2002). After sequencing the *SSU1* regions from vineyard strains, we discovered that the parent in Cross 1 has four of these repeats in the *SSU1* promoter, where the Cross 2 parent has only three. This raised the possibility that *SSU1* expression levels may influence the difference in grape juice growth of the two vineyard strains.

To test this hypothesis, we measured expression levels of *SSU1* transcript in oak and vineyard parents growing in rich medium. We found that strain KEH02580 had 64 times higher *SSU1* expression than the oak strain from Cross 1, whereas the other vineyard parent (M14) only shows 2.5 times higher expression than its companion oak strain (fig. 3*C*). To test the effect of *SSU1* expression, we cloned the vineyard allele onto a high-copy expression plasmid and introduced it into each of the strains. Overexpression of *SSU1* significantly increased the ability to grow in grape juice in all strains, but had the biggest effect in M14 (fig. 3*D*). Thus, higher expression of *SSU1* improves growth on grape juice and correlates with phenotypic variation in natural strains.

### Translocation at the *SSU1* Locus Provides a Genetic Barrier between Oak and Vineyard Strains

We were particularly interested in the fact that the *SSU1* allele is generated by a translocation, which could also present a genetic barrier between strains. Translocations are known to affect hybrid spore viability, for instance in interspecies hybrids of the *Saccharomyces* sensu stricto yeasts, and genomic rearrangements have been hypothesized to contribute to reproductive isolation (Ryu et al. 1998; Fischer et al. 2000; Delneri et al. 2003; Hou et al. 2014). The Chr 8:16 translocation is known to reduce spore viability in a vineyard strain mated to a lab strain (Hou et al. 2014), although the ecological relevance of this unnatural cross is unclear. We wondered if the Chr 8:16 translocation might contribute to the unexpectedly low spore viability between the oak and vineyard strains.

The spore viability of the oak × vineyard strains used for mapping ranged from 58% to 64% (table 1). Both vineyard strains harbored the reciprocal Chr 8:16 translocation, whereas the oak strains did not. In contrast, the spore viability for oak strains mated to one another was 90%. The same was true when a vineyard strain without the translocation was mated to either a vineyard strain or oak strain that also lacked the translocation. In contrast, the spore viability was reduced to 70% when a vineyard strain without the translocation was mated to a vineyard strain with the translocation. These results indicate that the spore viability of these strains correlated with the Chr 8:16 translocation genotype rather than the overall rate of polymorphism between strains.

**Table 1.** Translocation Lowers Spore Viability in Heterozygous Hybrids.

Cross	Strains Used in Cross	Spore Viability (%)
Oak (‐) × Vin (T), Cross 1	YPS163 × KEH02580	58
Oak (‐) × Vin (T), Cross 2	YPS1000 × M14	64
Oak (‐) × Oak (‐)	YPS163 × YPS1000	90
Oak (‐) × Vin (‐)	YPS163 × KEH02884	90
Vin (‐) × Vin (T)	KEH02580 × KEH02884	70

Note.—The spore viability for each cross is listed. (T) indicates strains harboring the Translocation and (‐) indicates strains without the translocation.

**Fig. 4.**
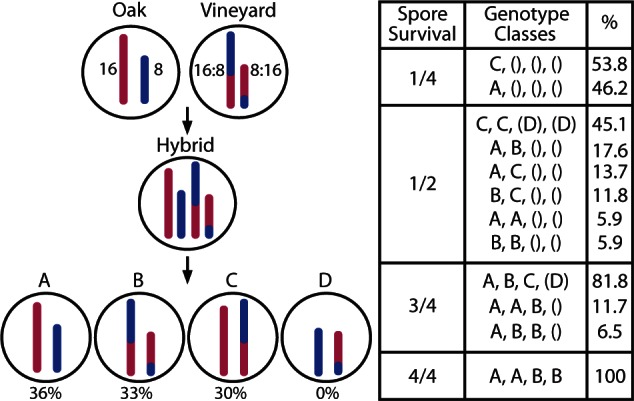
The Chr 8:16 translocation limits spore viability. The diagram to the left represents the Chr 8 and Chr 16 genotypes in the parent strains, the hybrid, and the four segregants (ignoring recombination for simplicity). The relative frequency of each genotype observed in 74–100 spores is listed below each genotype. The table to the right summarizes the observed tetrad-level genotypes classified by the number of viable spores (first column). () denotes dead spores that could not be genotyped and (D) denotes dead spores where genotype D is inferred. The last column (%) shows the percentage of each tetrad type per class.

The low spore viability could be caused by errors in chromosome pairing during meiosis, but it could also result if essential genes are lost in certain allelic combinations. To gain insight into the meiotic consequences of the translocation, we genotyped 75–100 tetrads from each of our crosses by diagnostic PCR to score the Chr 8:16 alleles. Sixty-nine percent of viable diploid cells harbored intact Chr 8 and Chr 16 without any translocation (Genotype A) or the two partner chromosomes participating in the reciprocal translocation (Genotype B) (fig. 4). In both of these cases, cells retain the full complement of yeast genes. A third diploid genotype (Genotype C) contained an intact Chr 16 and one of the translocated chromosomes containing most of Chr 8 and half of Chr 16 (Chr 16:8). Spores with this genotype have duplicated part of Chr 16, but are missing ten nonessential genes from the left tip of Chr 8 (fig. 4). Interestingly, the complementary genotype (in which half of Chr 16 is missing from the spores, Genotype D) was never found in a viable spore. These spores would lack a substantial portion of Chr 16, including 202 genes, 35 of which are essential (fig. 4). Genotypic analysis (through PCR) of spores confirmed these results and allowed us to determine the meiotic outcome of the crosses (fig. 4). These data confirm the expectation that the translocation can lead to significant meiotic defects, which in turn reduces spore viability. This also confirms the role of the reciprocal Chr 8:16 translocation in forming a genetic barrier between oak and vineyard strains.

### Translocation at the *SSU1* Locus Is Associated with a Cost in Oak-Associated Rapid Growth Rate

The features associated with the translocation are reminiscent of a so-called “magic trait” (Gavrilets 2004; Servedio et al. 2011; Chung et al. 2014) locus that underlies both divergent phenotypic selection and reproductive isolation. A common feature of such loci is that they provide divergent phenotypic cost–benefit in the two populations. Remarkably, we found that the translocation was detrimental to spores emerging from Cross 1 growing on control, no-stress media (fig. 2*E*). Oak strains have faster growth rate in standard medium, which is predicted to enrich for causal oak alleles in the oak × vineyard cross. Although the relevance of the selection conditions to the oak niche is not clear, the faster growth of oak-soil strains under these conditions is perhaps related to their advantage growing on low sugar or nonfermentable carbon sources in the wild (fig. 1*A* and *B*). These results show that the translocation acts as a magic locus that underlies both phenotypic divergence—by providing a benefit to a vineyard-associated phenotype and a cost to an oak associated trait—and reproductive isolation by reducing spore viability in hybrids.

## Discussion

### Niche-Related Phenotypes Distinguish Oak and Vineyard Populations

Our results show that oak strains and vineyard isolates are distinguished from each other both genetically and phenotypically, in a way that correlates with the unique features of their distinctive niches. The oak and vineyard environments likely impose very different stresses on the organisms thriving within them. We find that vineyard strains are better at surviving single- and multistress conditions that are characteristic of the domesticated vineyard environment. High osmolarity in grapes results from high sugar concentrations, and tartaric acid is the main weak-organic acid in grapes (Jackson 2000). Copper sulfate and other sulfur compounds are applied to vineyards as a fungicide and thus may have provided a selective pressure for adaptation in vineyard strains (Mortimer 2000; Besnard et al. 2001). Vineyard isolates also thrive in grape juice, in which many of the above stresses are applied simultaneously. This capability has likely been important in their adaptation to vineyard life. The ability of vineyard strains to survive these different stresses when each is applied individually suggests that cells adapted independently to deal with myriad features of a complex environment.

Oak strains are better at surviving a distinct set of stresses. The oak environment from which *S. cerevisiae* has been found is less well characterized than the vineyard environment. One thing that is clear is that the strains are unlikely to experience the abundance and range of sugars found in grapes. Others have suggested the oak environment may impose resource patchiness where nutrients are not as easy to come by Magwene et al. (2011); together, this may explain why oak strains have faster growth rates in rich media and are better at surviving low carbon conditions and on nonfermentable carbon sources. The oak strains are also more tolerant to a range of conditions that impose stress on the cell membrane and wall (including detergent, cell wall-damaging drugs, heat, and ethanol). The relevance of this is not clear but may reflect differences in cell wall composition. Interestingly, deletion of genes involved in cell wall signaling and structure, which generally increase sensitivity to cell-wall damage, provided an advantage under grape juice growth. Thus, the phenotypic distinction may reflect cell wall differences that may actually have been selected for in vineyard strains.

The distinguishing and ecologically relevant phenotypes could provide the ecological barriers necessary to keep oak and vineyard strains from invading each other’s niches. Although oak and vineyard strains are occasionally found in each other’s niche (Hyma and Fay 2013), vineyard strains greatly outnumber oak strains on grapes, whereas oak strains predominate in the soil and on the tree bark (Hyma and Fay 2013). Despite the ability to move between environments, our results suggest that these lineages are unlikely to thrive and reproduce in their nonadapted environment.

### The Genetic Basis of These Ecologically Relevant Phenotypes Is Complex

Using a bulk segregant mapping approach, we found that the genetic architecture of grape juice and Congo red tolerance is very complex. The genetic architecture of grape juice survival was different in the two crosses. The difference in *SSU1* expression levels between the two vineyard strains appears to contribute to the phenotypic difference, but other experimental differences (e.g., differences in selective pressure) may also explain the discrepancy. In contrast, there was significant overlap in the genetic architecture of Congo red resistance in the two crosses. In the case of Congo red resistance, almost half of the QTL were antagonistic, in that the allele of the sensitive parent provided an advantage. In contrast, the vineyard alleles provided the advantage for 77–100% of the grape juice QTL in both crosses, consistent with the notion of adaptive selection for grape juice tolerance (Orr 1998).

One of the major-effect loci contributing to growth in grape juice mapped to *SSU1*, whose importance was confirmed through our genetic and genomic screens. Importantly, the vineyard strains share the same protein-coding sequence of *SSU1*, indicating that the phenotypic difference is due to differences in *SSU1* expression. *SSU1* is a membrane sulfite/nitrite pump that exports excess sulfite produced during sulfate assimilation and methionine biosynthesis. The reason for its beneficial effect is not entirely clear: On the one hand, sulfur compounds are common antimicrobial agents applied to vineyards and industrial fermentations (Romano and Suzzi 1993); thus improved sulfite tolerance could have been selected for during wine-strain domestication. However, production of sulfide (via reduction of excess sulfite) is produced in response to low nitrogen levels or vitamin deficiency (Wang et al. 2003; Bohlscheid et al. 2007), which are known causes of sluggish fermentations (reviewed in Wang et al. 2003). These results suggest that higher sulfite export is not only beneficial to wine strains in industrial fermentations but could also contribute to growth of wild yeast on grapes in the natural vineyard niche. The frequency of the underlying Chr 8:16 translocation linked to the beneficial *SSU1* allele is also high in natural vineyard isolates, as half of wild vineyard strains studied here contain the translocation.

### Chr 8:16 Translocation Underlies Ecological and Genetic Barriers

The Chr 8:16 translocation that generates the beneficial *SSU1* allele was also implicated in the oak-specific trait of faster growth on rich medium, but with the opposite effect—the translocation provided a fitness cost. Although the ecological significance of this trait in the oak niche is unclear, these results indicate that the Chr 8:16 translocation is detrimental in a condition in which oak strains thrive. The Chr 8:16 translocation, therefore, underlies an ecological barrier between the oak and vineyard niches that could aid in keeping these two populations differentiated in nature. Strikingly, the Chr8:16 translocation also acts as postzygotic genetic barrier by reducing spore viability in F2 hybrids. Thus, the Chr 8:16 reciprocal translocation plays a dual role in providing a genetic barrier between the populations while reinforcing the ecological differentiation. Pleiotropic loci that have been linked to both phenotypic divergence and mate-choice behavior have been dubbed “magic” or “dual-trait” (Gavrilets 2004; Servedio et al. 2011; Chung et al. 2014) genes. These loci can greatly facilitate sympatric speciation, because the selection of divergent phenotypes simultaneously promotes reproductive isolation. However, the relative roles of these loci in speciation is unknown, as the genetics underlying most magic traits have not been identified (Chung et al. 2014). Our results show that the Chr 8:16 translocation could play a role in both ecological divergence and reproductive isolation between oak and vineyard populations. Consistent with this conclusion, two recent studies explore the potential for chromosomal rearrangements to initiate speciation in *S. cerevisiae* (Hou et al. 2014) and *S. paradoxus* (Charron et al. 2014).

In addition to the ecological and genetic barriers we present, differences in life-history traits between the populations likely contribute significantly to the dearth of oak × vineyard hybrids found in nature. Oak strains sporulate at the first signs of carbon shortage, whereas vineyard strains require extreme nutrient limitation before they produce gametes in the form of spores (Gerke et al. 2006, 2009; Magwene et al. 2011). Therefore, it is likely that strains from the two populations rarely encounter one another when they are both competent to mate. Together, these results highlight that multifaceted forces—including ecological differentiation, genetic barriers, and differences in life-history traits—underlie the reduced gene flow in oak and vineyard populations of yeast.

## Materials and Methods

### Phenotyping

Strains used are listed in supplementary table S1, Supplementary Material online. Phenotyping was carried out as in Kvitek et al. (2008) unless otherwise noted. Briefly, mid-log phase cultures were serial diluted onto agar plates containing appropriate stress (supplementary table S2, Supplementary Material online) and grown at 30**°**C for approximately 2 days. A semiquantitative growth score was assigned to each dilution spot, and scores were averaged across doses for each replicate. For liquid grape juice phenotyping, mid-log phase YPD (rich media, 10% yeast extract, 20% peptone, 20% dextrose) cultures were collected, washed in 30% white grape juice concentrate (Global Vintners Inc.), inoculated into fresh 30% white grape juice, and incubated 40 h in shaking incubator at 30**°**C. Data in figure 1 represent the average scores across all vineyard strains or all oak strains to assay differences between the two populations. The T-statistic distribution was calculated by randomly sampling from phenotype data 10,000 times. Raw phenotype scores are available in supplementary table S2, Supplementary Material online.

### Strain Crosses

We used a method similar to Ehrenreich et al. (2010) to generate large MAT**a** segregant pools. Each parent was transformed with an SGA-style cassette (Tong et al. 2001) containing the hygromycin resistance gene driven by the *MFA1* promoter (kindly supplied by C. Hittinger) and with homology to the homothallic switching endonuclease HO locus. Two versions of the cassette were created for selection of mated hybrids: One fused to the KanMX cassette and one fused to the NatMX cassette, for G418 resistance or nourseothricin resistance, respectively. Each cassette was amplified from a BY4741 strain, along with approximately 580 bp upstream and approximately 270 bp downstream sequence. These constructs were transformed into each of the parental strains, which were then sporulated and dissected to attain heterothallic MAT**a** and MAT**α** derivatives for mating. To avoid selection for either parental version of HO or MAT, four-way crosses of each parent with each cassette were performed, and selected diploids were pooled (see supplementary table S1, Supplementary Material online).

### Bulk Segregant Selection

Hybrids were sporulated on 1% potassium acetate plates for 10–14 days to allow at least 90% sporulation. Tetrads were collected by scraping sporulation plates and then treated with zymolyase (20 T, 0.5 mg/ml, final) at 30**°**C and vortexed to increase spore dispersal and to prevent mating (Parts et al. 2011). Spores germinated in liquid YPD for 3 h at 30**°**C, then MAT**a** segregants were selected in YPD with hygromycin for 6–7 generations. Pools of segregants (∼1 × 10^6^ cells) were collected then selected in 30% grape juice or YPD for 30 h or synthetic-complete medium with dextrose ± 1.4 mg/ml Congo red plates for 48 h. Cells were collected before and after selection and flash frozen in liquid nitrogen until DNA extraction.

### Sequencing

DNA was isolated from cell collections, using Qiagen Genomic Tip/100 G Kit. Libraries were prepared using Illumina TrueSeq DNA LT Sample Prep Kit v2 (#FC-121-2001) and sequencing on the Illumina HiSeq2000 (100 bp paired-end reads). Parental strains were sequenced to 16–20 × coverage, and bulks were sequenced to approximately 100 × coverage. Raw data were deposited in NIH (the National Institute of Health) SRA database under #SRP049418. Paired-end reads were aligned to the reference S288C genome (version R64) using Bowtie2 (Langmead and Salzberg 2012) with default settings and the –N parameter set to 1 to allow for mismatches. SNPs and indels were identified with GATK using base quality score recalibration, indel realignment, duplicate removal, and depth of coverage analysis (McKenna et al. 2010; DePristo et al. 2011). Default parameters were used except –mbq 25 to reduce false positive calls. Variants were filtered using the following suggested GATK criteria: QD < 2, FS > 60, MQ < 40.

### Bulk Segregant Analysis

To avoid potential mapping biases, an artificial reference genome was created for each cross: SNP differences common to both parents versus S288c were substituted in the reference sequence, whereas polymorphic sites between parents were replaced in the reference sequence with a third allele. This way, reads from both parents would have the same number of mismatches and thus avoid mapping biases. Reads were aligned to the appropriate reference using Bowtie2 (Langmead and Salzberg 2012) as above. A pileup was created using samtools (Li et al. 2009) at known SNPs. Allele counts at each SNP were calculated for bulk segregant analysis in MULTIPOOL (Edwards and Gifford 2012) in contrast mode with –N set to 5,000 comparing allele frequencies of the selected pool to allele frequencies of the starting pool before selection or to a control pool grown on YPD or SC medium where indicated. Similar results were attained with both mapping regimes (see supplementary table S3, Supplementary Material online). MULTIPOOL reports the logarithm of the odds (LOD) from a likelihood ratio test comparing models with and without a QTL. Before implementation, SNPs were filtered for coverage of at least 15×, presence of both parental alleles in both pools, and allele frequency between 0.1 and 0.9 to eliminate false signals in MULTIPOOL, resulting in 66–68,000 high-quality SNPs. To set a genome-wide threshold for peak detection, replicate unselected starting pools were run through the MULTIPOOL pipeline. We chose the maximum LOD score detected from control comparisons (10.68) as a conservative threshold for further analyses. We also performed bulk segregant analysis in no-stress YPD or SCD controls and identified very few significant QTL.

### Chemical Genomics Screen

A chemical genomic analysis of grape juice medium was performed as described in Piotrowski et al. (2015). Briefly, 200 µl cultures of a pooled collection of *S. cerevisiae* deletion mutants were grown in grape juice supplemented with amino acids (which improves lab-strain growth in grape juice [Harsch et al. 2010]), or a YPD control, for 48 h at 30 °C. DNA was extracted using the Epicentre MasterPure Yeast DNA purification kit. Mutant-specific barcodes were amplified with multiplex primers containing Illumina adapters (Smith et al. 2009). The barcodes of four replicates of each condition (grape juice vs. YPD) were sequenced using an Illumina HiSeq2500 Rapid Run platform. Differential abundance and significance were assessed for barcodes with at least ten reads using edgeR (Robinson et al. 2010). Functional enrichment was performed using FunSpec (Robinson et al. 2002). Fourteen of the 740 significant genes (FDR 0.05) fell under the 90% credible interval of grape juice QTL. Enrichment for genes under QTL peaks was assessed by randomly sampling 740 genes from the yeast genome and counting the frequency from 10,000 trials in which 14 or more genes fell under QTL peaks (supplementary table S5, Supplementary Material online).

### Cloning and Strain Construction for *SSU1* Validation

To generate the hemizygous hybrids, vineyard strain KEH02580 was transformed with the KanMX cassette and oak strain YPS163 was transformed with NatMX cassette with flanking homology to the HO locus and then sporulated and dissected to attain heterothallic MAT**a** and MAT**α** derivatives. The KEH02580 MAT**a** HO::KanMX and YPS163 MAT**α** HO::NatMX strains were transformed with HERP + HygMX (HERP1.1) cassette (Alexander et al. 2014) with homology to the *SSU1* locus. All integrants were verified by diagnostic PCR. Both hemizygous hybrids were mated as previously described. The wild-type *SSU1* hybrid and hemizygous hybrids were grown overnight in YPD, subcultured for four doublings, then inoculated into 40% grape juice for 24 h before final OD_600_ was recorded.

For overexpression constructs, the coding region of *SSU1* from vineyard strain KEH02580 was cloned by homologous recombination between the high-copy *TPI1* promoter and terminator onto a Nat-MX-marked CEN vector (pJH1_YDR050C). This vector or an empty vector control plasmid was used to transform the four parental strains from the two mapping crosses. Overnight cultures of each strain were grown in YPD + 50 µg/ml nourseothricin, subcultured for 1–2 doublings without drug (since strains grew very slowly in the presence of the drug), then inoculated into 40% grape juice without drug for 24 h before final OD_600_ was recorded. Note that these wild strains were very sensitive to nourseothricin. All strains with NatMX plasmids (empty and engineered) reached a higher OD in 40% grape juice after overnight growth in YPD + nourseothricin compared to strains grown overnight in YPD without nourseothricin.

### Translocation Genotyping and Phenotyping

Yeast strains were genotyped for the Chr 8:16 translocation using PCR primers that span the translocation breakpoint and through Sanger sequencing. Contigs were assembled using SeqMan software and aligned using ClustalW in MegAlign (DNASTAR Inc., Madison, WI). Genotyped vineyard strains were grown overnight in YPD, subcultured for four doublings, and inoculated into in 40% grape juice concentrate; growth was assayed by calculating the change in optical density (OD_600_) from the final optical density after 24 h of growth.

### RNA Sequencing

RNA-sequencing analysis was performed for all parental strains in YPD in the context of another unpublished study. Cells were harvested during log phase growth in YPD (OD_600_ ∼0.4), flash frozen in liquid nitrogen, and stored at −80**°**C until RNA extraction. Total RNA was extracted from yeast cells by the hot phenol method as previously described (Gasch 2002). Total RNA was DNase-treated at 37 °C for 30 min with TURBO DNase (Life Technologies AM2238), after which RNA was precipitated at −20 °C in 2.5 M LiCl for 30 min. rRNA depletion of the DNase-treated total RNA and subsequent cDNA library preparation were performed with ScriptSeq Complete Kit H/M/R (Epicentre BHMR1224), Index PCR Primers (Epicentre SSIP1234), and FailSafe PCR Enzyme Mix (Epicentre FSE51100). rRNA-depleted RNA was purified with a RNeasy MinElute Cleanup Kit (Qiagen 74204), and cDNA was purified with Axy Prep MAG PCR Cleanup beads (Corning MAG-PCR-CL-250). cDNA libraries were sequenced on the Illumina HiSeq2000, generating single-end 100 bp reads. Reads were processed with Trimmomatic (Bolger et al. 2014) and mapped to parent-specific reference genomes created by swapping S288C alleles with parental SNPs using Bowtie2 (Langmead and Salzberg 2012) with default settings and the –N parameter set to 1 to allow for mismatches. HTseq version 5.5 was used (Anders et al. 2014) to calculate read counts for each gene which were then normalized by calculating the reads per kilobase per million (RPKM) value for each gene RPKM. Log_2_(RPKM) values for *SSU1* were collected for each strain. Averaged biological duplicates were normalized to YPS163 for strain comparison.

### Spore Viability Analysis in Heterozygous Translocation Hybrids

Crosses and sporulations were performed as previously described. Sporulated hybrids were dissected on YPD plates and incubated 3 days before scoring spore viability and genotyping through PCR.

## Supplementary Material

Supplementary tables S1–S5 are available at *Molecular Biology and Evolution* online (http://www.mbe.oxfordjournals.org/).

Supplementary Data
