# Supplementary material for: Ecological and Genetic Barriers Differentiate Natural Populations of Saccharomyces cerevisiae
Source: Mol Biol Evol. 2015 May 6;32(9):2317–27. doi: 10.1093/molbev/msv112 (PMC4540968; doi:10.1093/molbev/msv112)
Supplement: Supplementary Data [file supp_32_9_2317__index.html]

Ecological and Genetic Barriers Differentiate Natural Populations of Saccharomyces cerevisiae — Ecological and Genetic Barriers Differentiate Natural Populations of Saccharomyces cerevisiae — Supplementary Data 

# Ecological and Genetic Barriers Differentiate Natural Populations of *Saccharomyces cerevisiae*

## Supplementary Data

files

- Supplementary Data - xlsx file
- Supplementary Data - xlsx file
- Supplementary Data - xlsx file
- Supplementary Data - xlsx file
- Supplementary Data - xlsx file
